# Ingestibility and Formulation Quality of Lansoprazole Orally Disintegrating Tablets

**DOI:** 10.1155/2016/6131608

**Published:** 2016-12-01

**Authors:** Sumio Chono, Megumi Matsui, Katsuki Nakamura, Ryoya Kasai

**Affiliations:** ^1^Division of Pharmaceutics, Hokkaido Pharmaceutical University School of Pharmacy, Hokkaido, Japan; ^2^Keihan Hospital, Osaka, Japan

## Abstract

*Objectives*. We evaluated the ingestibility and formulation quality of one branded (formulation A) and five generic (formulations B, C, D, E, and F) lansoprazole orally disintegrating (OD) tablets.* Methods*. Ingestibility, including the oral disintegrating time, taste, mouth feeling, and palatability, was examined by sensory testing in healthy subjects. Formulation qualities, including salivary stability, gastric acid resistance, and intestinal dissolution behavior, were examined.* Results and Discussion*. The oral disintegration time of formulation F (52 s) was significantly longer than that of other formulations (32–37 s). More than 90% of subjects did not experience bitterness with formulations A, E, and F, whereas 50% of subjects felt rough and powdery sensations with formulations B, C, and D. More than 80% of subjects suggested that formulations A, E, and F had good palatability. Ingestibility was different between formulations. OD tablets consist of enteric granules containing lansoprazole, which is unstable in gastric acid. Enteric granules of each formulation were stable in artificial saliva and gastric juice. No differences were observed in dissolution behaviors among the formulations, indicating that the formulation quality of the formulations was almost equivalent.* Conclusions*. This study provides useful information for selecting branded or generic lansoprazole OD tablets for individualized treatments.

## 1. Introduction

Orally disintegrating (OD) tablets are rapidly dissolved or disintegrated in the oral cavity [1], with patients able to take these tablets with or without water. Therefore, OD tablets are convenient for patients in any situation, including patients with limited water intake, those who have difficulty obtaining water because of a busy lifestyle, and those with decreased deglutition function [2].

Lansoprazole OD tablets are frequently used for treating gastric ulcers, duodenal ulcers, and reflux esophagitis in the clinical setting [3–5]. In addition to Takepron® OD tablets as a branded formulation [6], several generic formulations are also available. Lansoprazole OD tablets are compressed tablets consisting of enteric granules and other additives because lansoprazole is unstable in gastric acid and is loaded into enteric granules. These characteristics are common to both branded and generic formulations [6–11].

Many generic formulations are currently in clinical use. Because a variety of lansoprazole OD tablets are available in the market, physicians and pharmacists have difficulty in selecting suitable formulations for individual patients [12]. The cost price from wholesalers is not an optimal criterion for selecting formulations, warranting selection on the basis of scientific assessments of ingestibility and formulation quality. Ingestibility indexes that directly influence the medication compliance of lansoprazole OD tablets include disintegration time in the oral cavity, taste, mouth feeling, and palatability. In addition, salivary stability, gastric acid resistance, and intestinal dissolution behaviors are important formulation quality indexes. However, little is known regarding the ingestibility and formulation quality of lansoprazole OD tablets.

Here, ingestibility and formulation quality were assessed in one branded and five generic lansoprazole OD tablets. Briefly, sensory tests were performed on healthy subjects to assess the oral disintegration time, taste, mouth feeling, and palatability, and stabilities under salivary and gastric acid conditions and dissolution behaviors under intestinal conditions were examined.

## 2. Methods

### 2.1. Materials

The tested lansoprazole OD tablets are presented in Table 1.

### 2.2. Sensory Testing

Sensory testing was performed with approval from the Hokkaido Pharmaceutical University president (number 12-04-001) following an examination of the protocols by the study ethics committee. Informed consent was obtained from all subjects, and 50 healthy volunteers (21 men and 29 women, 23–64 years) were included in the study.

Sensory testing was performed as previously described [13]. In brief, subjects put one OD tablet on the tongue, picked it up with the tongue and upper jaw (disintegration initiation time point), and then orally disintegrated the samples. The time taken for the complete disintegration of samples was recorded at the end of the disintegration. The disintegrating time of OD tablets in oral cavities was measured by subjects using a stopwatch. Disintegrated OD tablets were then immediately removed with saliva and thoroughly washed from the oral cavities with water. Finally, subjects answered a questionnaire (Table 2) to record the taste, mouth feeling, and palatability.

All subjects were blinded to the names and manufacturers of OD tablets and were tested with the tablets at 1 h intervals on the same day.

### 2.3. Stability Tests in the Artificial Saliva and Gastric Juice

One OD tablet was added into artificial saliva (5 mL, Saliveht® Aerosol, Teijin Pharma Limited, Tokyo, Japan) or artificial gastric juice (30 mL, pH 1.2, 1st fluid in disintegration test of the Japanese Pharmacopoeia 16th edition [14]), followed by incubation at 37°C for 2 min (saliva) or 15 min (gastric juice). Subsequently, samples were immediately centrifuged at room temperature (650 ×g for 10 min) to separate enteric granules from artificial saliva or gastric juice. Separate enteric granules were dispersed into 1 mol/L NaOH solution (30 mL) and kept at 37°C for 15 min. Then, enteric granules were completely crushed using ultrasound mastax (Powersonic Model 50, Yamato Scientific Co., Ltd., Tokyo, Japan), followed by centrifugation at room temperature (650 ×g for 10 min). Supernatants were diluted 40 times with 1 mol/L NaOH solution, and dilutions were mixed with CH_3_OH (1/1, v/v). The absorbance of these samples at 284 nm was measured using a spectrophotometer (UV1280, Shimadzu Corporation, Kyoto, Japan), and the drug retention (%) in enteric granules was calculated.

### 2.4. Dissolution Test in Artificial Intestinal Juice

A dissolution test based on the Japanese Pharmacopoeia 16th edition [15] was performed using the Toyama dissolution tester (Toyama Sangyo Co., Ltd., Osaka, Japan). The test solution was a 2nd fluid (900 mL, pH 6.8) which acted as an artificial intestinal juice with the testing temperature at 37°C ± 0.5°C. The turnover of the paddle was 100 rpm. At each time point, a 5 mL aliquot of the eluate was collected, and the test solution (5 mL) was immediately supplemented. Collected eluates were filtered using a membrane filter (pore size: 0.45 *μ*m) and then mixed with CH_3_OH (1/1, v/v). The absorbance of these samples at 284 nm was measured using a spectrophotometer as described above, and the drug dissolutions (%) from enteric granules were calculated.

### 2.5. Statistical Analysis

Statistical differences were identified using Tukey's honestly significant difference test, and changes were considered significant when *p* < 0.05.

## 3. Results

### 3.1. Oral Disintegration Time

**Figure 1:**
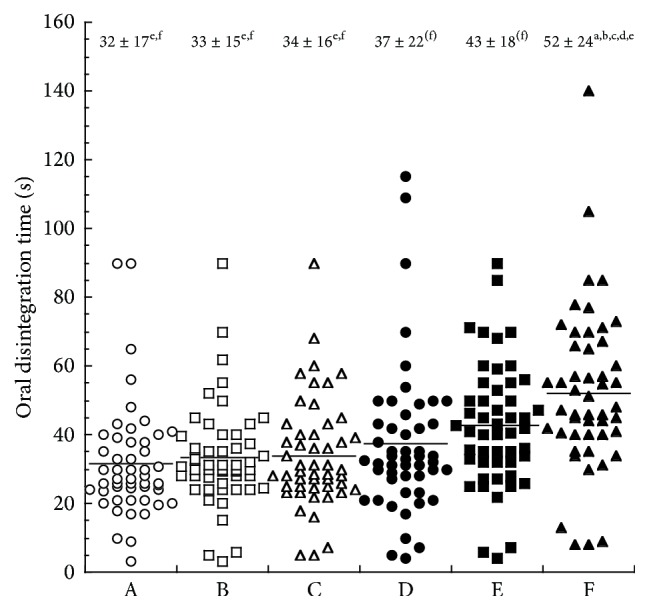
Oral disintegration times of lansoprazole OD tablets. (a) *p* < 0.05 versus A, (b) *p* < 0.05 versus B, (c) *p* < 0.05 versus C, (d) *p* < 0.05 versus D, (e) *p* < 0.05 versus E, and (f) *p* < 0.05 versus F. Data are presented as means ± standard deviations (SD; *n* = 50). Each symbol is individual data and bars are mean values.

The oral disintegration times of lansoprazole OD tablets are shown in Figure 1. The oral disintegration time of formulation F (52 ± 24 s) was significantly longer than that of formulations A (32 ± 17 s), B (33 ± 15 s), C (34 ± 16 s), D (37 ± 22 s), and E (43 ± 18 s).

### 3.2. Taste, Mouth Feeling, and Palatability

**Figure 2:**
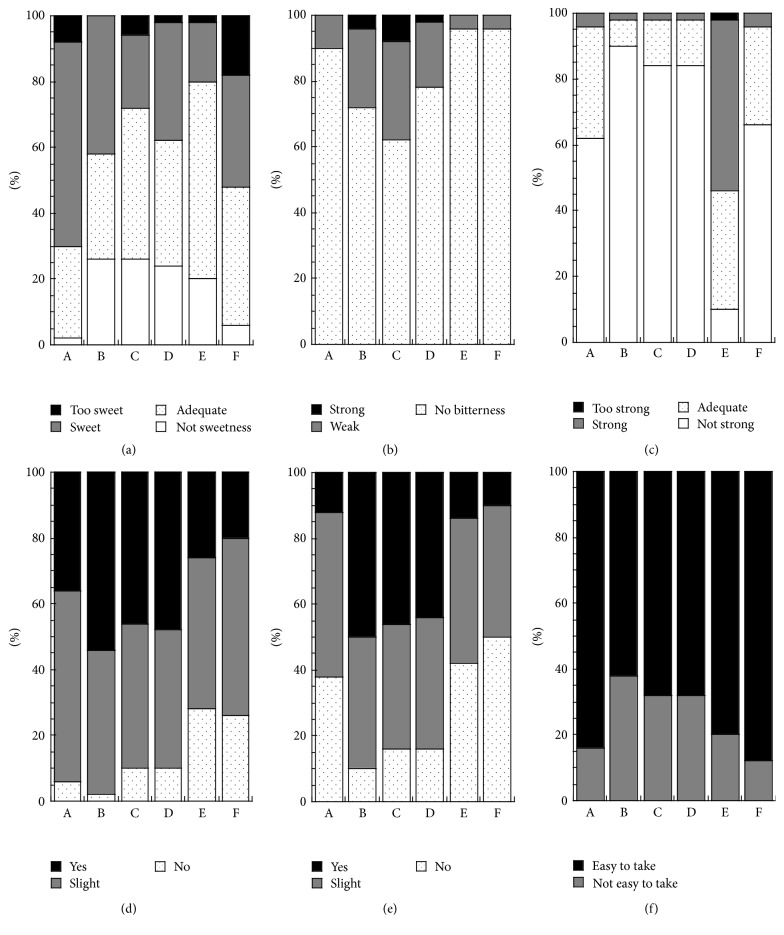
Taste, mouth feeling, and palatability of lansoprazole OD tablets. (a) Sweetness; (b) bitterness; (c) mentholated taste; (d) granular/rough; (e) powdery; and (f) palatability.

Taste, mouth feeling, and palatability of OD tablets are shown in Figure 2. In terms of taste and mouth feeling, more than 90% of subjects did not feel bitterness with formulations A, E, and F (panel (b)), whereas approximately 50% of subjects felt rough and powdery sensations with formulations B, C, and D (panel (d) and (e)). Each formulation was generally considered to be sweet (panel (a)). Characteristically, many subjects felt the mentholated taste in formulation E (panel (c)). Finally, more than 80% of subjects described formulations A, E, and F as easy to take, indicating good palatability (panel (f)).

### 3.3. Stability in Artificial Saliva and Gastric Juice

**Figure 3:**
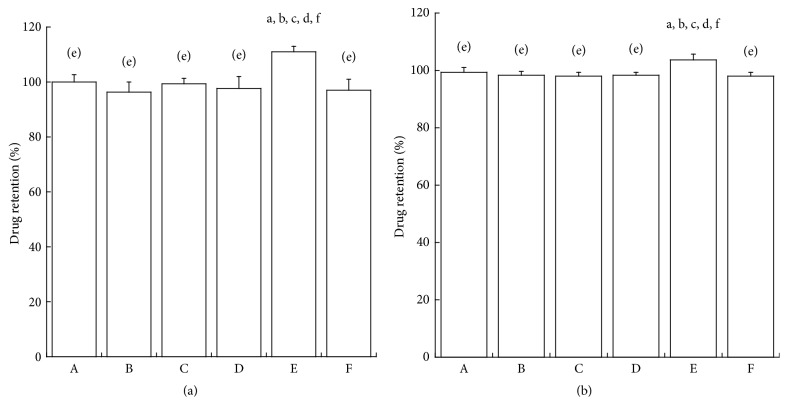
Stability of lansoprazole OD tablets in artificial saliva (a) and artificial gastric juice (b). Data presented are mean ± SD (*n* = 5).

Stability of lansoprazole OD tablets in artificial saliva and gastric juice is shown in Figure 3. Drug retention (%) in enteric granules exposed to artificial saliva and gastric juice in each formulation was approximately 100%.

### 3.4. Dissolution in Artificial Intestinal Juice

**Figure 4:**
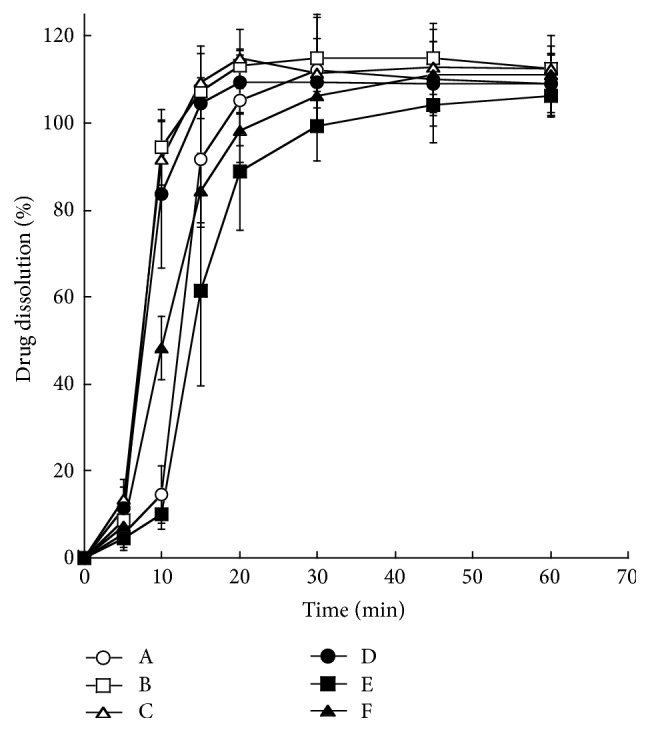
Dissolution behaviors of lansoprazole OD tablets in artificial intestinal juice. Data are presented as mean ± SD (*n* = 5).

Dissolution behaviors of lansoprazole OD tablets in artificial intestinal juice are shown in Figure 4. Although some differences were observed in the initial increase of the dissolution rate between formulations, complete dissolutions (approximately 100%) were observed in each formulation within 20–30 min.

## 4. Discussion

The oral disintegration time of formulation F was the longest (Figure 1), indicating that the velocity of immersion wetting [16] of formulation F with saliva is slower than other formulations, presumably reflecting differences in the additives and compression pressure. Patients reportedly feel no major stress from tablets with disintegration times less than 60 s [17], indicating that all the present formulations had suitable oral disintegration times. Taste, mouth feeling, and palatability differed among formulations (Figure 2), reflecting differences in the additives. The palatability of lansoprazole OD tablets was in agreement with subject-reported sweetness and bitterness, level of mentholated taste, and granular/rough and powdery sensations. We have demonstrated that taste, mouth feeling, and palatability of tamsulosin hydrochloride OD tablets also differ among branded and generic formulations [12]. In this study, we performed sensory tests in healthy subjects. The amount of saliva may influence the evaluation of ingestibility in sensory tests. According to a previous report [18], both age and disease status influence salivary secretion. Thus, further detailed examinations that account for saliva secretion based on age and disease status are required.

Enteric granules of each formulation were stable in artificial saliva and gastric juice (Figure 3). Consequently, it is suggested that enteric granules of each formulation are stable in the oral cavity and normally show gastric acid resistance. The major difference in dissolution behaviors between formulations was not observed in artificial intestinal juice, and complete dissolutions were observed in each formulation within 20–30 min (Figure 4), suggesting that enteric granules of each formulation release lansoprazole in intestines normally. Referentially, the dissolution of each preparation in artificial gastric juice (pH 1.2) was approximately 1%-2% for 60 min (data not shown). Though dissolution tests do not guarantee bioequivalence among formulations [19], the large biological nonequivalency among the six aforementioned formulations can be avoided.

## 5. Conclusions

In this study, ingestibility, including oral disintegration time, taste, mouth feeling, and palatability, of lansoprazole OD tablets was evaluated, and formulation qualities, including salivary stability, gastric acid resistance, and intestinal dissolution behavior, were examined. The ingestibility among the six formulations differs, although the formulation qualities were almost equivalent. The present study provides useful information for selecting branded or generic lansoprazole OD tablets for individualized treatments.

## Figures and Tables

**Table 1:** Lansoprazole OD tablets.

	OD tablets (Manufacturer)	Lot number
A	Takepron® OD tablets, 15 mg (Takeda Pharmaceutical Company Ltd., Osaka, Japan)	OJ344
B	Lansoprazole OD tablets, 15 mg “JG” (Nihon Generic Co., Ltd., Tokyo, Japan)	203840
C	Lansoprazole OD tablets, 15 mg “DK” (Daiko Pharmaceutical Co., Ltd., Kawagoe, Japan)	AR01
D	Lansoprazole OD tablets, 15 mg “Taiyo” (Teva Pharma Japan Inc., Nagoya, Japan)^*∗*^	AY1305
E	Lansoprazole OD tablets, 15 mg “Towa” (Towa Pharmaceutical Co., Ltd., Osaka, Japan)	A057
F	Lansoprazole OD tablets 15 mg “Nichi-Iko” (Nichi-Iko Pharmaceutical Co., Ltd., Toyama, Japan)	GI1401

^*∗*^Current name is lansoprazole tablets, 15 mg “Teva.”

**Table 2:** Questionnaire and sensory testing options.

Questionnaire	Options
Sweetness	Too sweet
	Sweet
	Adequate
	Not sweetness

Bitterness	Strong
	Weak
	No bitterness

Mentholated taste	Too strong
	Strong
	Adequate
	Not strong

Granular/rough	Yes
	Slight
	No

Powdery	Yes
	Slight
	No

Palatability	Easy to take
	Not easy to take
